# Idiosyncratic Responses of High Arctic Plants to Changing Snow Regimes

**DOI:** 10.1371/journal.pone.0086281

**Published:** 2014-02-11

**Authors:** Sabine B. Rumpf, Philipp R. Semenchuk, Stefan Dullinger, Elisabeth J. Cooper

**Affiliations:** 1 Department of Conservation Biology, University of Vienna, Vienna, Vienna, Austria; 2 Institute for Arctic and Marine Biology, University of Tromsø, Tromsø, Troms, Norway; 3 Institute of Interdisciplinary Mountain Research, University of Vienna, Vienna, Vienna, Austria; 4 Department of Arctic Biology, University Center in Svalbard, Longyearbyen, Svalbard, Norway; 5 Department of Geosciences and Natural Resource Management, University of Copenhagen, Copenhagen, Capital Region of Denmark, Denmark; 6 Vienna Institute for Nature Conservation and Analyses, Vienna, Vienna, Austria; WSL Institute for Snow and Avalanche Research SLF, Switzerland

## Abstract

The Arctic is one of the ecosystems most affected by climate change; in particular, winter temperatures and precipitation are predicted to increase with consequent changes to snow cover depth and duration. Whether the snow-free period will be shortened or prolonged depends on the extent and temporal patterns of the temperature and precipitation rise; resulting changes will likely affect plant growth with cascading effects throughout the ecosystem. We experimentally manipulated snow regimes using snow fences and shoveling and assessed aboveground size of eight common high arctic plant species weekly throughout the summer. We demonstrated that plant growth responded to snow regime, and that air temperature sum during the snow free period was the best predictor for plant size. The majority of our studied species showed periodic growth; increases in plant size stopped after certain cumulative temperatures were obtained. Plants in early snow-free treatments without additional spring warming were smaller than controls. Response to deeper snow with later melt-out varied between species and categorizing responses by growth forms or habitat associations did not reveal generic trends. We therefore stress the importance of examining responses at the species level, since generalized predictions of aboveground growth responses to changing snow regimes cannot be made.

## Introduction

Snow depth is one of the drivers governing growing season length in the Arctic [1]–[4]. It also affects winter soil temperatures through thermal insulation of soil and vegetation, thus controlling nutrient turnover rates and availability [5], as well as soil moisture during the early growing season [3], [4]. These are all factors influencing plant growth [6], and thus the carbon sink capacity of arctic ecosystems which are nutrient, moisture, and light (i.e. snow-free season) limited [7]–[10]. Moreover, the amount of available plant biomass and temporal patterns of plant phenology may have cascading effects on many aspects of the ecosystem, such as pollinators, herbivores, pathogens, pests [7], [11], as well as on the energy balance of the ecosystem and its albedo.

Changes of temperature and precipitation regimes driven by climatic change will impact arctic and alpine snow cover and are hence expected to have profound direct (e.g. melt-out date and temperature sums) and indirect effects (e.g. nitrogen mineralization rates) on arctic and alpine ecosystems [4], [5], [12]. The Intergovernmental Panel on Climate Change (IPCC) emphasizes an increase of winter precipitation and winter temperatures in the Arctic in its Fourth Assessment Report [13], and these findings are backed up by their 2013 report (see http://igloo.atmos.uiuc.edu/IPCC/). However, the magnitude and direction of snow cover changes are not easily predictable since they strongly depend on how precipitation sums are distributed across the winter season and, in particular, the partitioning into snow and rain fractions [14].

Depending on these factors, two possible scenarios are conceivable. (1) Due to rising temperatures in winter, the fraction of precipitation falling as rain could increase and hence reduce snow cover depth and duration leading to a longer growing season [2], [4], [10]–[12], [15]–[18]. (2) Temperatures remain low enough during periods of maximum precipitation to increase winter snow depth with subsequent later snowmelt, leading to a shortened growing season [4], [19], [20]. In recent years, an increase of spring temperatures [13], [21] and an increasing frequency of extreme rain events during winter [22] resulted in a general trend towards an earlier snowmelt in the high Arctic [13], [21], [23]. But the alternative scenario remains plausible, too: increasing cloud cover during the light season could lead to a delay of snowmelt even with reduced snow depth [4], [24]. In addition, climatic forecasts for the Arctic are usually on a large scale and do not account for potentially pronounced regional differences. As an example, the climate of Svalbard, where this study is situated, differs from many other high arctic localities by its maritime climate and is influenced by the gulf stream, resulting in relatively warm winters, cold summers and strong temperature fluctuations during all seasons [14], [25].

Over and above these uncertainties about the future of arctic snow regimes the likely ecosystem consequences of a changing snow pack are also variable. Studies in other arctic regions suggest an increase of net primary production (NPP) following a prolonged growing season, i.e. after early snowmelt [9], [22]. However, Pop et al. [16] reported that some plant species were unable to increase or may even decrease their growth in response to an early onset of the growing season into a thermally less favorable time of the year. Gamon et al. [8] even reported a general decline of productivity at his study site. Such an earlier advance could lead to tissue damage by spring frosts and cold winds after snowmelt, since plants lose their frost hardiness during the onset of growth. A decrease in plant growth following delayed snowmelt [27] is assumed to be due to the constraining effect of a short growing season and reduced temperature sums on biomass accumulation [4]. Additionally, a deeper snow pack leads to colder soils after snowmelt [20], [28] but warmer soils during winter [20], [29], leading to a negative carbon balance for some species (especially ridge plants) that greatly increase respiration rates during warmer winters [4]. On the other hand, a deeper and prolonged snow cover (a) shelters plants from spring frosts and cold winds (see above), (b) entails warmer soils in winter, potentially increasing nitrogen (N) mineralization and thereby improving nutrient availability in spring [5], [18], [28], [30], [31], (c) results in moister soils during the early growing season [18], [20], [24], [28], [32], (d) postpones the onset of plant growth to a climatically more suitable period [18], [33], and (e) protects plants from exposure to cold winter air in the case of mid-winter snow melt due to extreme weather events [34], [35], all factors which could potentially alter ecosystem productivity and plant growth.

In the light of these opposing scenarios and conjectures, generic predictions of how the future snow pack will affect arctic plant growth remain difficult. In this study we therefore searched for potential generic trends by exploring how a delayed and an earlier melt might affect the aboveground growth of several common high arctic plant species. To do so, we experimentally manipulated snow depth and thus melt dates in the field by means of snow fences (increasing snow depth, delaying melt) and shoveling (decreasing snow depth, advancing melt), calculated how these manipulations changed the cumulative temperature sums received by the experimental plots, and studied growth responses of target plants to the treatments by measuring plant sizes weekly throughout a full growing-season.

We hypothesized that 1) melt out date and temperature sums would affect aboveground plant size (early melt and high temperature sum would increase plant size in relation to *Normal*; the opposite for late melt), and 2) patterns of response may be observed at the general (all species together), growth form (graminoids, herbs, shrubs) and habitat association (snowbeds, ridges) level [4], [7].

## Materials and Methods

### Experimental setup

Fieldwork was conducted in Adventdalen (78°10′N, 16°06′E), Svalbard, Norway, throughout summer 2011, from 1 May until 12 September. Annual mean air temperature during 2002–11 (Longyearbyen airport) was −3.7°C, and average temperatures of the coldest month (March), the warmest month (July) and annual mean precipitation were −13.5°C, 7.2°C and 177 mm, respectively [41]. The mean air temperature during June, July and August (JJA) during 2002–11 was 5.8°C and 6.3°C during 2011. The study year can thus be considered an average year in that five of the 10 preceding years had average JJA temperatures close to 6.3°C. Similar considerations hold for accumulative temperature sums (thawing degree days, TDD) and accumulative precipitation during the same period (see Table S1 for details). During 2008 to 2012, our group reported winter warming events in 2010 and 2012 [35], resulting in snow removal and subsequent extreme low soil temperature spikes during mid-cold-season which interfered with flower abundances of some species. No such warming event or similar spiking of soil temperature occurred during the winter preceding the present study, so we thus exclude the possibility of frost damage of our study species during the cold-season 2010–2011. Air temperatures after melt-out remained positive throughout the growing-season in all snow regimes, i.e. no growing-season freezing events were observed in 2011.

The experimental setup is based on Morgner et al. [20]. We used nine of the twelve existing snow fences (1.5 m tall and 6.2 m long) which were established in autumn 2006, distributed over an area of approximately 1.5 km×2.5 km and grouped into blocks of three fences (each 200×200 m) that were erected at least 500 m apart from each other to account for heterogeneity of the landscape. The fences were established perpendicular to the main winter wind direction, such that snow transported by wind accumulated behind the fences (leeside) due to turbulences. Behind each fence, two subplots of 75×75 cm were selected: one in the area of the deepest snow (approx. 150 cm, thereafter called *Deep*), and another one in the area of intermediate snow depth (60–100 cm, thereafter called *Medium*), both representing a climate scenario that predicts a moderate to pronounced delay of snowmelt and hence a shortened growing season. To account for a climate scenario that predicts less snow in favor of rain and hence an earlier melt-out we designated a subplot next to each fence on a small windblown ridge that melted out naturally earlier than average (snow depth: 1–5 cm, in the following named *Shallow*) and another one on which the snow was manually removed on 1 May (snow depth 10–35 cm, in the following named *Removed*). In contrast to the other snow regimes, *Removed* subplots were newly established in autumn 2010. We compared these regimes with current conditions in an unmodified *Normal* subplot for each fence, in an area representative for most of the valley's snow depth, approx. 10–35 cm. The average melt-out dates during 2008–2012 were 24 May in *Shallow*, 2 June in *Normal*, 12 June in *Medium*, 19 June in *Deep* [35]. Since not all treatments (i.e. snow regimes) could be realized at each fence the experiment was based on a total of 37 subplots: four *Removed*, eight *Shallow*, nine *Normal*, seven *Medium* and nine *Deep*. The low number of *Removed* subplots is due to marker stick removal by reindeer during wintertime.

The land on which the field site is situated belongs to Store Norske Spitsbergen Grubekompani [42] and the fieldwork permit for this study was obtained from this company, the Governor of Svalbard [43] (reference number 2006/00803-3a.*521*) and the Longyearbyen Lokalstyre [44] (reference number 2009/401-2) and no protected species were sampled.

### Abiotic measurements

Before snowmelt, all subplots were observed every second day and were defined as snow free when 50% of their area had melted out. In each block (i.e. set of three fences) one data logger was installed (Tiny Tag Plus 2, Gemini Data Loggers, UK) which recorded soil temperatures hourly at approximately two centimeters below the soil surface from 4 September 2007 in *Normal* and *Deep*, and from 1 June 2010 in *Removed*, *Shallow*, and *Medium*. Daily average soil temperatures of each logger were used for the entire block. Daily average air temperatures at two meters above the ground were taken from the new weather station of the University Centre in Svalbard in Adventdalen [45] around six kilometers west of the study site in the same valley. Soil moisture of thawed soil was measured weekly using a hand held Theta probe ML2X (delta-T devices, Cambridge, UK) 4–5 times at each of at six subplots per snow regime, spanning the site to account for the vegetative and geographic heterogeneity.

From the temperature measurements we calculated nine different temperature variables for each subplot, three from air and six from soil measurements (Table 1): these variables represent either the number of days with a mean temperature above 0°C or the cumulative temperature sums above this threshold (thawing degree days, TDD) since either melt-out dates or 1. May, i.e. the date on which the snow was shoveled away on *Removed* subplots. In addition, we also calculated the number of days with a mean temperature above 5°C. TDD and number of days until the day of each growth measurement were then matched with the recorded plant size.

**Table 1:** Overview of the nine calculated cumulative temperature variables (temperature sums) based on daily average temperatures.

Air or soil temperature	Beginning of record	Threshold	Used value
Air	Melt-out date	0°C	Temperature in °C
Air	Melt-out date	0°C	Number of days
Air	Melt-out date	5°C	Number of days
Soil	Melt-out date	0°C	Temperature in °C
Soil	Melt-out date	0°C	Number of days
Soil	Melt-out date	5°C	Number of days
Soil	1. May	0°C	Temperature in °C
Soil	1. May	0°C	Number of days
Soil	1. May	5°C	Number of days

Melt-out dates were recorded for each subplot.

### Biotic measurements

All biotic measurements were based on the ITEX manual [40] and were carried out from 13 June until 8 September 2011 at weekly intervals. For those study species not mentioned in the ITEX manual we adapted protocols following those of similar species or growth forms. We chose eight of the most common species of the study site as target species, including deciduous and evergreen shrubs, graminoids and perennial forbs, as well as snowbed and ridge species: *Alopecurus magellanicus*, *Bistorta vivipara* (syn. *Polygonum viviparum*), *Cassiope tetragona*, *Dryas octopetala*, *Luzula arcuata* subsp. *confusa*, *Pedicularis hirsuta*, *Salix polaris* and *Stellaria crassipes* (Table 2; nomenclature according to The Flora of Svalbard [46]).

**Table 2:** Overview of the species-specific parameters per individual, growth form and habitat association.

Species	Species specific parameter	Growth form	Habitat association
*Alopecurus magellanicus*	Sum of leaf lengths (from ligule to leaf tip)	Graminoid	Snowbed
*Bistorta vivipara*	Sum of leaf areas (calculated as ellipse based on leaf length and width)	Forb	Snowbed
*Cassiope tetragona*	Annual increment of one shoot	Evergreen shrub	Snowbed
*Dryas octopetala*	Sum of leaf lengths of one shoot (excluding petiole)	Evergreen shrub	Ridge
*Luzula arcuata*	Sum of leaf lengths (from soil surface to leaf tip)	Graminoid	Ridge
*Pedicularis hirsuta*	Plant length (from soil surface to uppermost leaf)	Forb	Snowbed
*Salix polaris*	Sum of leaf lengths of one shoot (excluding petiole)	Deciduous shrub	Snowbed
*Stellaria crassipes*	Plant length (from soil surface to uppermost leaf)	Forb	Ridge

As soon as a subplot had melted out, or individuals of a given species were visible, one randomly chosen individual or ramet per species was selected. For *Salix* four individuals per subplot were chosen intending to include a female and a male specimen in the study. Plant size was measured with an electronic caliper with an accuracy of 0.1 mm. Only green parts (assumed to be photosynthetically active) were measured. If the marked individual got lost due to grazing or wind removal of the marker, a new randomly chosen individual or ramet nearby was marked and observed from then on and treated as a replicate in order to avoid loss of data. 43.5% of the recorded individuals or ramets were followed throughout the complete season.

For each species different measurements were taken according to their morphology (Table 2). For *Alopecurus*, *Dryas*, *Luzula* and *Salix* the summed length of all leaves per shoot (in mm) excluding the petiole or ligule (where applicable) was used. For *Bistorta* the length and width of each leaf was used to calculate the leaf areas as ellipses and single leaf values were then summed for each individual. For *Pedicularis* and *Stellaria*, plant length/height was measured from soil surface until the uppermost leaf. For *Cassiope*, the growth increment of a shoot of the year was used since the insertion of the youngest leaf on the caulis was not easily visible.

### Statistical analyses

Since the data was collected in a hierarchically organized experimental set up, we used linear mixed-effects models for analysis. We assumed a unimodal relationship between time and plant size since plants usually do not grow cumulatively throughout the vegetation period but with a peak during early to mid-season, followed by a decline of live plant tissue due to senescence and leaf drop. In the analysis, we hence fitted a second order polynomial of each of the nine different temperature variables to the size measurements (Table 1) of each species separately, with random effects for fence area, fence (i.e. plot), subplot and individual. We selected the best among the nine temperature models per species based on the Akaike Information Criterion (AIC), and the one with the lowest AIC was chosen. We then sequentially removed each term of the selected full model and compared which of the reduced models was the best fitting one (i.e. with the lowest AIC) and then used that to predict temperature sum needed until (and magnitude of) maximum plant size for each species and snow regime by determining the peaks of the hump-shaped functions.

Treatment effects on (1) melt-out dates, (2) the different temperature variables, and (3) the average size of the species throughout the season were also evaluated by means of mixed effects models with the same group structure as defined above. Potential heteroscedasticity was considered for as much grouping levels as possible (i.e. parameter estimation algorithms converted). With respect to plant size, these analyses were conducted for every species separately as well as for all species together to test for generic trends. In the latter case, we scaled all species-specific size measurement to a common range between 0 and 1 and added growth form and habitat association as predictor variables, as well as species identity as an additional (highest) group variable. Each model was then sequentially reduced and the one with the lowest AIC selected. All analyses were conducted with R [47] using the packages nlme [48] and lattice [49].

## Results

Melt-out dates of the *Normal* regime differed significantly from all other snow regimes (Table 3). Snow was manually *Removed* on 1 May, *Shallow* regime was snow free on 30 May, *Normal* on 4 June, *Medium* on 12 June, *Deep* on 16 June; thus the onset of the snow free period varied by up to 46 days between regimes. Soil moisture was high (55–70%) immediately following snowmelt, and dropped throughout the growing season (to 30–50% at end August). Soils in *Deep* and *Medium* were moister- and those in *Shallow* were drier- than Normal (see Figure S1). Manual snow removal led to a pooling of water in the subplot *Removed* due to an influx of melting water from surrounding areas (personal observation), and this gave rise to higher soil moisture during the growing season than in the *Normal* or *Shallow* regimes (Figure S1).

**Table 3:** Estimates of treatment effects on melt-out dates.

	Melt-out dates			TDD		
	Effect ± sd	t-value	p-value	Effect ± sd	t-value	p-value
Intercept (Normal)	155±1.0			272.7±6.7		
Removed	−34±0.9	−39.9	<0.001	45.7±11.6	3.95	0.003
Shallow	−5±0.7	−6.9	<0.001	10.7±9.3	1.16	0.253
Medium	8±0.6	14.1	<0.001	−26.4±9.6	−2.74	0.009
Deep	12±0.5	26.2	<0.001	−38.9±9.0	−4.35	<0.001
df	182			6180		

Melt-out dates in days of year (doy) and on TDD (thawing degree days, i.e. positive air temperature sums in °C). Effect values other than the intercept (here: Normal treatment) are deviations from the latter. Normal = unmanipulated snow cover; Removed = snow removal on 1. May; Shallow = naturally early snowmelt; Medium = intermediately increased snow; Deep = maximally increased snow. Given are standard deviation (sd), t- and p-values and degrees of freedom (df) of the model.

Thawing degree days of air temperatures since melt-out (TDD) was the temperature variable that explained plant size for all species best, due to its lowest AIC. For *Dryas octopetala* only the TDD of air since 1 May was a better fitting variable, but TDD since melt-out was still a highly significant predictor for this species' size (AIC difference: 6). For consistency, we have therefore used TDD since melt-out in all subsequent analyses of all species. The TDD after melt-out of individual subplots depends on melt-out dates only, since air temperatures were derived from one single weather station. Consequently, this variable differed between treatments in parallel to melt-out dates although the difference between *Normal* and *Shallow* subplots was not statistically significant (Table 3), most probably because of low air temperatures in spring. However, no other variable calculated from our temperature measurement series was able to explain temporal plant growth patterns in a similarly consistent way across species.

### Growth pattern

#### All species

Only *Shallow* had an effect on the aboveground growth when regarding all species together and led to decreased plant sizes (Table 4). Both growth form and habitat association did not remain in the model after model selection and comparison of AIC, hence there were no generic aboveground growth patterns detectable - of the different growth forms or habitat associations- resulting from variable TDD or melt-out dates.

**Table 4:** Estimates of treatment effects on plant sizes of all species throughout the growing season.

	Effect ± sd	t-value	p-value
Intercept (Normal)	0.22±0.03	6.56	
Deep	−0.02±0.02	−0.88	0.379
Medium	−0.02±0.02	−1.12	0.263
Removed	0.02±0.02	0.75	0.456
Shallow	−0.06±0.02	−3.54	<0.001
df	294		

Effect values other than the intercept (here: Normal treatment) are deviations from the latter. Species-specific size measurements were scaled to a common range between 0 and 1. Given are standard deviation (sd), t- and p-value and degrees of freedom (df) of the model. Normal = unmanipulated snow cover; Removed = snow removal on 1. May; Shallow = naturally early snowmelt; Medium = intermediately increased snow; Deep = maximally increased snow.

#### Species-specific overview

The species-specific results are summarized in Table 5. The *Deep* regime led to decreased growth of *Cassiope* and *Salix* but increased of *Bistorta* and *Dryas*. *Medium* led to reduced plant sizes of *Cassiope*, *Pedicularis* and *Stellaria* compared to *Normal* but to increases in *Alopecurus*, *Bistorta* and *Dryas*. *Shallow* snow led to a reduced growth of *Cassiope*, *Luzula* and *Pedicularis* but increased the size of *Stellaria*. The *Removed* regime generally had a different response than *Shallow*, and this treatment favoured two species (*Dryas* and *Luzula*) but led to smaller plants of *Cassiope* and *Pedicularis*.

**Table 5:** Significant increases (+) or decreases (−) in plant size compared to *Normal* due to treatment.

	*Removed*	*Shallow*	*Medium*	*Deep*
*Salix polaris*				*−*
*Cassiope tetragona*	*−*	*−*	*−*	*−*
*Pedicularis hirsuta*	*−*	*−*	*−*	
*Luzula arcuata*	*+*	*−*		
*Stellaria crassipes*		*+*	*−*	
*Alopecurus magellanicus*			*+*	
*Dryas octopetala*	*+*		*+*	*+*
*Bistorta vivipara*			*+*	*+*

#### *Alopecurus magellanicus*

**Figure 1:**
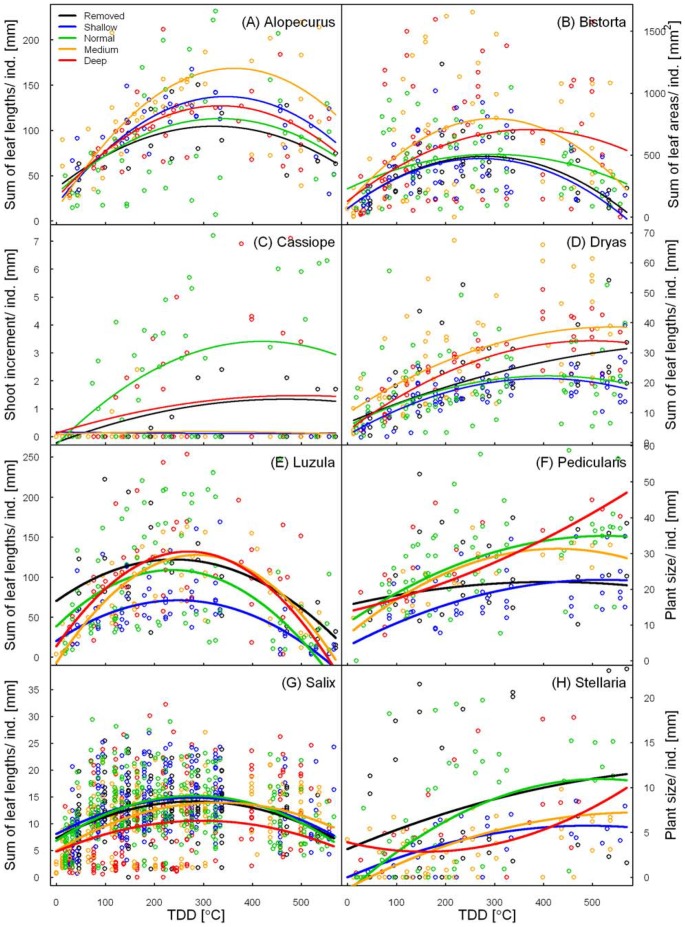
Relationship between TDD (cumulative sum of thawing degree days, i.e. positive air temperature sums) and plant sizes. a) the average sum of leaf lengths of all *Alopecurus magellanicus* individuals; b) the average sum of leaf areas of all *Bistorta vivipara* individuals; c) the average annual shoot increment of all *Cassiope tetragona* individuals; d) the average sum of leaf lengths of all *Dryas octopetala* individuals; e) the average sum of leaf lengths of all *Luzula arcuata* individuals; f) the average plant size of all *Pedicularis hirsuta* individuals; g) the average sum of leaf lengths of all *Salix polaris* individuals; h) the average plant size of all *Stellaria crassipes* individuals. Coefficients are derived from a quadratic linear mixed-effect model, separated by treatments. Normal = unmanipulated snow cover; Removed = snow removal on 1. May; Shallow = naturally early snowmelt; Medium = intermediately increased snow; Deep = maximally increased snow. Outliers are not shown for better visualization. Note: The green (living) plant size decreases at end of season (highest TDD) due to senescence and leaf-drop.

The summed leaf lengths of *Alopecurus* were only significantly higher in *Medium* than in *Normal* plots, namely by 50% (Table 6). In all treatments similar amounts of TDD were required to reach peak sizes and range between 322°C (*Removed*) and 363°C (*Medium*; Figure 1a).

**Table 6:** Estimates of treatment effects on plant sizes throughout the growing season, as well as peak size and number of thawing degree days needed to reach peak size.

Species	Treatment	Effect ± sd	t-value	p-value	df	Peak size	TDD peak
***Alopecurus magellanicus***	Intercept (Normal)	84.4±11.4				112.9	334.0
	Removed	14.1±13.3	1.06	0.291		104.5	322.4
	Shallow	15.2±10.4	1.45	0.148		137.2	345.2
	Medium	42.1±9.9	4.25	<0.001		168.4	362.8
	Deep	19.1±10.0	1.91	0.058	173	127.0	334.9
***Bistorta vivipara***	Intercept (Normal)	360.5±60.6				505.4	296.0
	Removed	23.8±73.0	0.33	0.745		489.1	280.2
	Shallow	11.3±63.7	0.18	0.859		471.6	271.9
	Medium	238.3±63.2	3.77	<0.001		793.1	297.6
	Deep	344.3±60.0	5.73	<0.001	295	705.1	370.1
***Cassiope tetragona***	Intercept (Normal)	2.32±0.29				3.41	419.6
	Removed	−1.37±0.39	−3.53	<0.001		1.34	466.7
	Shallow	−2.13±0.31	−6.78	<0.001		0.16	0.0
	Medium	−1.89±0.32	−5.93	<0.001		0.18	295.1
	Deep	−0.84±0.29	−2.91	0.004	177	1.47	494.9
***Dryas octopetala***	Intercept (Normal)	18.2±2.2				22.2	410.2
	Removed	5.1±2.0	2.51	0.013		31.3	570.0
	Shallow	−0.8±1.7	−0.45	0.653		21.3	399.0
	Medium	11.0±1.9	5.96	<0.001		38.5	542.0
	Deep	6.9±1.9	3.69	<0.001	288	33.9	497.6
***Luzula arcuata***	Intercept (Normal)	77.4±10.9				108.9	236.6
	Removed	20.5±8.7	2.35	0.020		122.0	240.1
	Shallow	−23.8±7.1	−3.37	<0.001		71.0	248.5
	Medium	−7.9±7.6	−1.05	0.297		127.7	287.1
	Deep	11.2±7.0	1.60	0.112	261	131.9	269.0
***Pedicularis hirsuta***	Intercept (Normal)	29.6±2.2				34.9	526.6
	Removed	−4.5±1.8	−2.46	0.016		22.0	413.3
	Shallow	−12.4±1.5	−8.02	<0.001		22.6	520.8
	Medium	−5.7±1.9	−3.04	0.003		31.3	428.8
	Deep	−0.4±2.2	−0.17	0.867	112	47.0	570.0
***Salix polaris***	Intercept (Normal)	12.1±0.7				15.2	281.3
	Removed	0.3±0.7	0.51	0.611		14.1	283.0
	Shallow	0.6±0.6	1.08	0.283		14.8	280.3
	Medium	−0.2±0.6	−0.40	0.693		13.9	329.4
	Deep	−1.3±0.6	−2.34	0.020	324	10.5	296.2
***Stellaria crassipes***	Intercept (Normal)	6.9±1.4				10.9	515.7
	Removed	3.1±1.9	1.60	0.112		11.5	570.0
	Shallow	4.4±1.9	2.36	0.020		5.7	489.9
	Medium	−3.4±1.7	−1.98	0.050		7.2	570.0
	Deep	−1.8±2.0	−0.89	0.373	148	10.0	570.0

Effect values other than the intercept (here: Normal treatment) are deviations from the latter. Given are standard deviation (sd), t- and p-value and degrees of freedom (df) of the model. Modeled maximal plant sizes and TDD (thawing degree days, i.e. positive air temperature sums in °C) to reach the maximum size are based on the model shown in Table 3, and are shown as values for each treatment, rather than deviation from the Normal treatment. Normal = unmanipulated snow cover; Removed = snow removal on 1. May; Shallow = naturally early snowmelt; Medium = intermediately increased snow; Deep = maximally increased snow. Measures for plant species: average sum of leaf lengths (in mm) for *Alopecurus magellanicus*, *Dryas octopetala*, *Luzula arcuata*, *Salix polaris*; average sum of leaf areas (in mm^2^) for *Bistorta vivipara*; average annual shoot increment (in mm) for *Cassiope tetragona*; average plant sizes (in mm) for *Pedicularis hirsuta*, *Stellaria crassipes*.

#### *Bistorta vivipara*

The sum of *Bistorta* leaf area increased significantly behind fences, by 95% and 66% in *Deep* and *Medium*, respectively (Table 6, Figure 1b). Individuals in *Deep* reached their full size later, i.e. at higher levels of TDD, and they preserved their maximum biomass for a longer period of time. In regimes becoming snow-free earlier than *Normal*, the size of *Bistorta* individuals did not significantly differ from those of *Normal*; maximal plant sizes also required about the same temperature sums.

#### *Cassiope tetragona*

All treatments lowered the shoot increment of *Cassiope* compared to *Normal* (Table 6, Figure 1c). Most individuals in *Medium* and *Shallow* subplots did not grow at all throughout the season, so reduced the shoot increment to 82% and 92% of *Normal*, respectively. In the *Deep* and *Removed* regimes, shoots of *Cassiope* grew to only 36% and 59% of *Normal* lengths, and the modeled maximum plant sizes were only 57% and 61% of those of *Normal*, respectively.

#### *Dryas octopetala*

All treatments apart from *Shallow* increased leaf lengths and peak sizes for *Dryas* in comparison to *Normal* (Table 6). Treatment effects were most pronounced in *Medium* where plants grew 60% larger than *Normal*, and the estimated maximum plant size was almost 75% larger, followed by *Deep* (38% increase in total leaf length, 53% larger peak sizes), where the species grew fastest after snowmelt (Figure 1d). In the *Removed* treatment, a leaf length increase of 28% was recorded compared to *Normal*.

#### *Luzula arcuata*

Growth responses of *Luzula* to differing snow regimes were rather inconsistent (Table 6, Figure 1e). In *Removed*, average plant size was significantly increased by 27% compared to *Normal*, whereas *Shallow* significantly reduced the species' growth by almost the same extent (24%). In parallel, plants reached a 12% higher maximum leaf length in *Removed*, but a 35% lower maximum in *Shallow* regime. Shortening the growing season did not affect the species' full-season leaf length significantly.

#### *Pedicularis hirsute*

Earlier as well as later snowmelt had a negative effect on the average plant size of *Pedicularis*. However, no significant effect was found in *Deep*, maybe because of scarcity of data for this treatment (Table 6). The full-season plant size was decreased by 15% in *Removed*, 42% in *Shallow* and 19% in *Medium*. The individuals in *Removed* regime had a peculiar temporal growth pattern: they grew rapidly in the beginning and then their size hardly changed throughout the rest of the season, whereas plants in other snow regimes grew more steadily (Figure 1f).

#### *Salix Polaris*

Only a very late snowmelt (*Deep* regime) had a significantly negative effect on the average total length of *Salix* leaves and reduced it by 11% compared to *Normal* conditions (31% in terms of maximum length; Table 6). In the *Medium* treatment leaves were smaller than in *Normal* during their early growth phases, but an accelerated growth later in the season compensated for this disadvantage (Figure 1g). Peak sizes were hence similar to those of *Normal* individuals, but plants required higher TDD sums to reach their maximum size (329.4 in *Medium*, compared to 281.3 in *Normal*). Earlier snowmelt (*Removed* and *Shallow* regimes) did not affect the leaf length significantly.

#### *Stellaria crassipes*

The average length of *Stellaria* decreased with a delayed and increased with an advanced snowmelt, although this trend was only significant for the intermediate regimes - with a decrease of 49% (3.5 mm) in *Medium* and an increase of 64% (11.3 mm) in *Shallow* compared to *Normal* (Table 6, Figure 1h). Furthermore, the modeled maximal sizes and TDD to reach this size suggest that individuals in *Removed* and *Medium* grew until the end of the season, but not those in *Normal* and *Shallow*. However, these trends are based on scarce data and we hence consider the regression and maximal plant sizes of individuals in *Deep* as unreliable.

## Discussion

Cumulative air temperature (TDD) since snowmelt was found to be the best predictor of aboveground plant size. Surprisingly, the cumulative soil temperatures at our site did not describe the plant size as well as the air temperatures 6 km away, and from our data it is not possible to tease out whether this is due to insufficient geographical resolution of microclimatic thermal variation [61] or whether it is the air temperature driving plant growth. This could be tested by installing air and soil temperature loggers at each subplot, which we did not do here. Our findings, however, do indicate that the use of data from nearby climate stations may be useful for up-scaling into areas without detailed plot-level temperature data.

Generalizations regarding aboveground growth responses of all vascular plants to changes in the snow regimes studied are hard to make. Responses were species-specific and not grouped to growth forms or habitat associations (snow bed vs. ridge species). Analysis of data across all eight species indicated just one trend; lower aboveground plant size on ridges (i.e. *Shallow* snow regime). However, even this response was not common to all species, but driven by a relatively strong effect of *Shallow* on some of them. The detected species-specific variation of aboveground growth responses to different snow regimes is probably a consequence of niche specializations of each individual species. Chapin and Shaver [36] concluded that growth of different species is limited by species-specific adaptations and competition, probably leading to observed individualistic responses to environmental manipulations which could not be explained by growth form, as was also the case in our study. No single environmental factor seems to be able to explain growth limitations of a whole community.

Only some of our species followed the hypothesized response, i.e. an earlier growing season start and higher temperature sum increased plant size, and later melt/lower temperature sum reduced plant size, compared to *Normal* snow regime. However, the responses to deeper snow/later melt might be driven by factors other than growing season length: e.g. deeper snow leads to increased soil moisture, cooler soils and higher nutrient availability after snowmelt [3], [4], [18], [20], [24], [28], [30], [31], [32]. Any change of snow depth and melt-out dates hence entails a trade-off between positive and negative alterations to the abiotic environment in terms of the microclimate that roots and shoots experience, as well as of nutrient and water availability. Since there are species-specific differences in growing season and winter temperature demands, nutrient and water requirements do not strictly parallel simultaneous changes in these conditions and resource supply rates result in varied response patterns [36]. For instance, plants that grew better following enhanced winter snow depth might have partly responded to increased nutrient supply [5], [37], [50], [51]. Indeed, in the same experiment our group observed a threefold increase of ammonium (NH_4_^+^) and a small increase of nitrate (NO_3_^−^) in soils under deep snow [29]. Soil moisture was enhanced in deeper snow treatments, especially during the first part of the growing season [20] and became similar to that of *Normal* regime by the end of the season [29] (see also Figure S1). Moisture limited species might therefore benefit from deepened snow particularly in the beginning of the season, in the period of fastest growth.

An intermediate increase of snow cover in *Medium* can thus lead to a beneficial trade-off for nutrient-, season length- and temperature limited high arctic vegetation [10], [18], [24], [39], [52] and enable greater growth by providing enhanced moisture and nutrient availability [29] with a season length maintained above a critical threshold. This was most likely the case for *Alopecurus magellanicus*, *Bistorta vivipara* and *Dryas octopetala* in our study. A positive growth response to controlled nutrient addition in Ny-Ålesund, Svalbard, was also reported for *B. vivipara* [50], [53] and *D. octopetala* [51] (although not in Robinson et al. [53] in the same study). In contrast, we detected a negative effect of a moderately increased snow pack on the growth of *Cassiope tetragona*, *Pedicularis hirsuta* and *Stellaria crassipes*, which was what we expected when only taking season length into account. These species were probably not able to benefit enough from improved nutrient/soil moisture supply [29] and were negatively affected by the delayed snowmelt. Schimel et al. [5] suggested that species with dynamic root systems might be able to benefit from a nutrient flush in spring caused by deep winter snow before N immobilization occurs shortly after. However, as a semi-parasite and cryptophyte, *Pedicularis* might have slow growing roots, and the same could hold for the slow growing dwarf shrub *Cassiope*. That could explain both species' inability to benefit from snow fence induced nutrient flushes [29], and their negative response to the later snowmelt and shortened growing season. Additionally, as shown by Havström et al. [54] *Cassiope* did not respond to fertilizer addition but to summer temperatures in relatively cold Svalbard with opposite responses in the warmer sub-arctic lowlands of Abisko, Sweden. They thereby suggest a co-limitation of temperature and nutrient availability for that species, where growing temperature demands have to be fulfilled before enhanced nutrients can be utilized.

The highly variable, species-specific responses of arctic plants to changes in snow pack are a generic finding, which holds even if our experimental manipulations have some associated caveats. Indeed, the experimental treatments resulted in specific combinations of growing season length, microclimate during (early) growth and nutrient and water [29]. These conditions are unlikely to become fully realized under natural conditions following climate change, mainly because some treatments had artificial side effects that were only due to our manipulations. This is particularly true for the *Removed* treatment, which may have gained additional nutrients washed in with the extra melt water from surrounding areas, which might, together with extra moisture, have contributed to the enhanced growth of *Dryas* and *Luzula*. Second, climate change may also ameliorate early season climatic conditions [7], which might enhance the potential beneficial effect of an earlier melting date. In contrast to the assumption for arctic ecosystems [12], [26] a naturally earlier snowmelt and thus a longer growing season (*Shallow*) only resulted in an increased biomass for *Stellaria* - in terms of average but for not for peak plant sizes. *Cassiope*, *Luzula* and *Pedicularis* even had lower average and peak sizes in *Shallow* compared to *Normal*. We assume that these results are due to a shift of the onset of growth and thus of the determination of leaf set towards climatic conditions which are cooler than expected under a future warmer climate [18], [33], [55] and a more likely exposure of dehardened plant tissue to spring frosts and cold winds [11], [16], [55], as well as reduced thermal insulation during winter. On the other hand, the *Shallow* snow cover at these sites might be representative for future average conditions and expose aboveground tissues to fluctuating air temperatures causing dehardening during winter warm spells, followed by exposure to cold winter temperatures. This potentially harms overwintering meristems and reduces growth in the following season, as has been observed in *Vaccinium myrtillus* in the sub-Arctic during winters with low snow cover [56], [57]. Under these conditions, growth as observed in *Normal* snow regime might only occur during growing seasons following exceptionally stable winters without dehardening periods. In addition, recent research has demonstrated that in contrast to aboveground growth, sexual reproduction of alpine and arctic plants may suffer severely even from episodic mild frost events during anthesis [58] which will likely still occur under warmer average spring temperatures. At least in the longer term many plants are hence at risk from shallower snow and earlier melt-out.

While our hypothesis 2 was rejected regarding the uniformity of species in their growth response to an earlier or later snowmelt, we could accept hypothesis 1 as cumulative temperature sums since the date of snow melt were indeed found to be a highly significant predictor of the size of arctic plants. In fact the critical role of temperature sums for plant life in cold-limited climates is not too surprising [37], [50], [59]. As a consequence, the size of the plants depended less on the length of the season but more on the overall input of warmth during the snow-free period. For instance, a shortened growing season could be compensated by higher air temperatures and vice versa. This suggests that the response of plants in the future to altered snow pack characteristics will strongly depend on the climatic conditions following melt-out.

Grouping species into plant functional types (PFTs) according to their physiology rather than growth form might simplify the analyses of community responses to environmental perturbations [38], such as grouping to 1) periodic species with a genetically fixed growing period, and 2) aperiodic species with a growing period constrained by external factors (e.g. *Bistorta* [7]) as summarized by Wookey et al. [60]. In the case of *Cassiope* and *Pedicularis* the majority of individuals steadily grew until the end of season under most treatments, irrespective of accumulated temperature. These two species could therefore be categorized as aperiodic functional types, which grow until environmental conditions become unfavorable. However, all other observed species (*Alopecurus*, *Bistorta*, *Dryas*, *Luzula*, *Salix* and *Stellaria*) stopped growing after a given temperature sum. These could therefore be categorized as periodic species with growth limited by internal factors. This also suggests that these species are unlikely to benefit greatly from an earlier or warmer spring and longer potential growing season, and that is likely to have knock-on effects for grazing herbivores relying on these plants as forage. However, we emphasize that we only measured one aspect of growth, while little is known about the dynamics of belowground or stem growth, which might well continue until after leaf growth ceased.

## Conclusions

We demonstrated that air temperature sums since melt-out best explained aboveground growth for all species in most snow regimes. We conclude that the response of high arctic plants to climate-driven changes in snow regimes is highly species-specific, and thus it is very important to study several common high arctic species instead of only one. Some species were very responsive (e.g. *Bistorta vivipara*), others were highly resistant (e.g. *Salix polaris*); still others seemed to respond negatively to any changes (e.g. *Cassiope tetragona*), and this may explain their occurrence in the landscape.

Certain species (*Luzula arcuata*, *Salix polaris*) were unaffected by a moderate increase in snow depth. In contrast, a deep snow pack reduced the growth of certain species (*Cassiope tetragona*, *Salix polaris*), most likely due to reducing the growing season length to under a threshold level; yet this regime increased the growth of other species (*Bistorta vivipara*, *Dryas octopetala*), possibly due to 1) increased soil moisture in early summer and 2) enhanced mineralization rates during winter resulting in increased nutrient availability during summer [29]. In the *Shallow* snow regime that melted five days earlier than *Normal*, all plants (except *Stellaria crassipes*) were smaller, possibly as a result of decreased protection from cold winter air temperatures or start of growth early in the spring time whilst temperatures were still cold. The *Removed* regime enhanced the growth of *Luzula arcuata* and *Dryas octopetala* in contrast to their response to shallow snow, indicating that enhanced moisture and possibly in-washed nutrients (due to the influx of melt water from surrounding areas) were contributing factors.

Many of our studied species showed a periodic growth pattern, i.e. plant size increases stopped after a certain cumulative temperature was obtained. This is important especially regarding forage availability for herbivores as it indicates that a warmer growing season may simply lead to an earlier peak instead of an increase in biomass. A spatially patchy environment in summer would enable a range of phenological stages and plant sizes to be available for foragers, instead of all plants reaching their peak size simultaneously. In this way, it would spread the occurrence of peak plant size over a longer duration in the summer. By contrast, removal of late lying snow-beds would diminish this patchiness in the foraging environment, which may be to the herbivores' disadvantage.

We point out that this study presents an imperfect simulation of future conditions as it does not account or control for (1) realistic changes in temperature, especially during spring time, and (2) the confounding effects of altered moisture and nutrient supplies. Thus we recommend future studies to investigate these issues in more detail, for example by arranging snow fence experiments along ambient temperature gradients and measure nutrient and moisture supply rates as covariates. Nevertheless, climate change and its consequences for arctic snow regimes will certainly affect temperature conditions during the growing season and winter as well as nutrient and moisture supply simultaneously. The plant species studied responded differently to the various snow regimes, indicating that a changing climate is likely to result in a shift in species composition. Indirectly, such a shift in species composition will probably affect community, and finally ecosystem productivity.

### Data availability

According to field-specific standards the data of this study is not deposited publicly but can be obtained directly from the authors upon request.

## Supporting Information

Figure S1 **Soil moisture measured at the experimental site in Adventdalen throughout the growing season in 2011.**
(TIFF)

Table S1 **Climatic variables recorded in Adventdalen, Svalbard for June, July and August, 2002–2011; mean air temperatures (C), Thermal Degree Days (TDD, cumulative in C) and accumulative precipitation (mm).**
(DOCX)
