# Supplementary figures and images for: Idiosyncratic Responses of High Arctic Plants to Changing Snow Regimes
Source: PLoS One. 2014 Feb 11;9(2):e86281. doi: 10.1371/journal.pone.0086281 (PMC3921108; doi:10.1371/journal.pone.0086281)

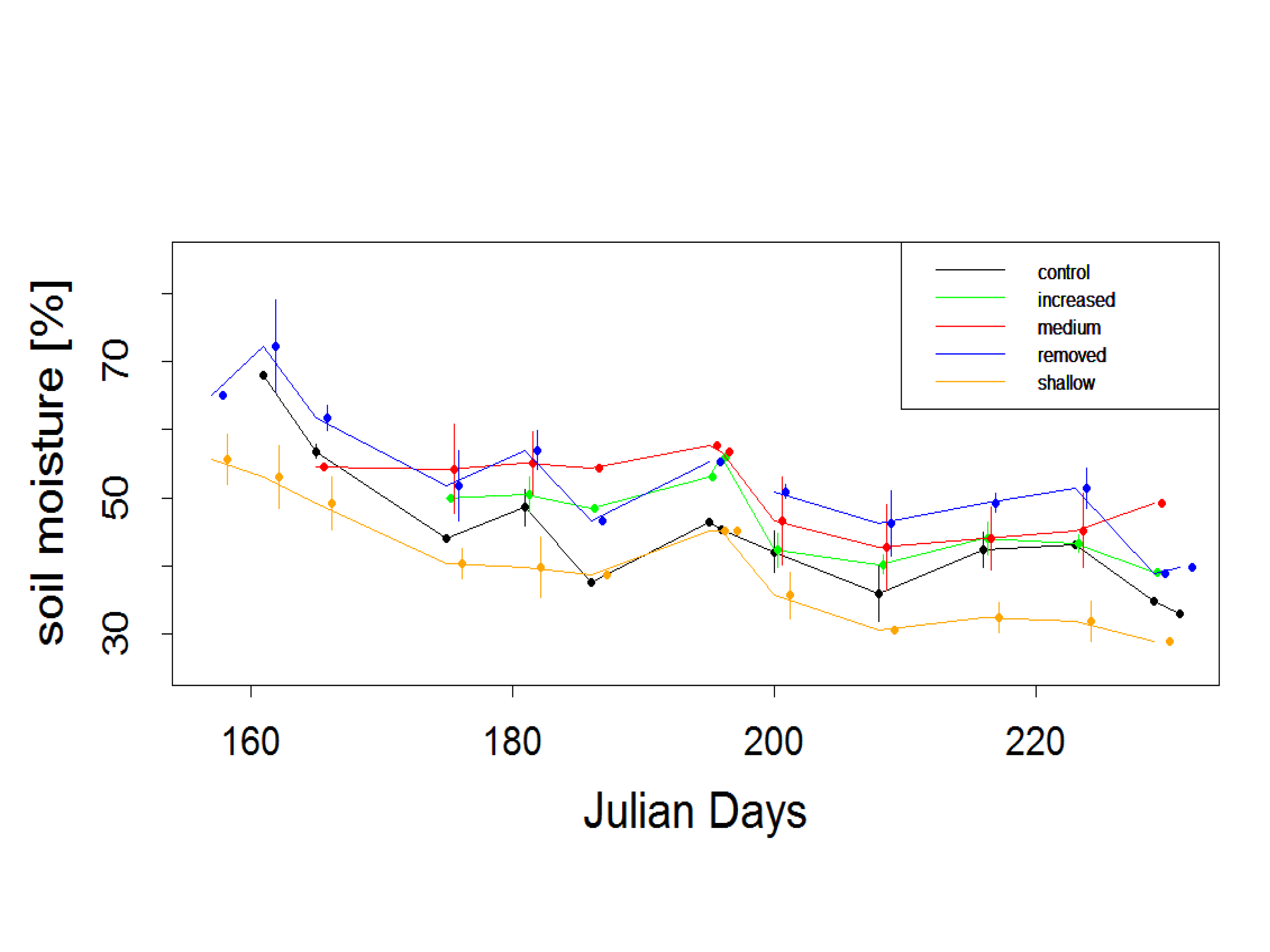

Supplement: Figure S1 — Soil moisture measured at the experimental site in Adventdalen throughout the growing season in 2011. (TIFF) [file pone.0086281.s001.tiff]
