# Supplementary material for: Idiosyncratic Responses of High Arctic Plants to Changing Snow Regimes
Source: PLoS One. 2014 Feb 11;9(2):e86281. doi: 10.1371/journal.pone.0086281 (PMC3921108; doi:10.1371/journal.pone.0086281)
Supplement: Table S1 — Climatic variables recorded in Adventdalen, Svalbard for June, July and August, 2002–2011; mean air temperatures (C), Thermal Degree Days (TDD, cumulative in C) and accumulative precipitation (mm). (DOCX) [file pone.0086281.s002.docx]

|  |  |  |  |
| --- | --- | --- | --- |
| Year | JJA mean temp  (C) | JJA TDD  (C) | JJA accum.precip.  (mm) |
|  |  |  |  |
| 2002 | 6.3 | 575.9 | 10.2 |
| 2003 | 5.6 | 511.1 | 9.5 |
| 2004 | 5.6 | 516.0 | 18.3 |
| 2005 | 6.3 | 577.6 | 14.0 |
| 2006 | 6.3 | 578.4 | 15.3 |
| 2007 | 6.4 | 592.0 | 5.3 |
| 2008 | 4.9 | 450.0 | 12.5 |
| 2009 | 5.6 | 519.5 | 6.9 |
| 2010 | 5.0 | 461.3 | 7.5 |
| 2011 | 6.3 | 575.8 | 6.2 |
| average | 5.8 | 535.7 | 10.6 |
